# Morphometric Analysis of the Mandibular Canal, Anterior Loop, and Mental Foramen: A Cone-Beam Computed Tomography Evaluation

**DOI:** 10.3390/ijerph18073365

**Published:** 2021-03-24

**Authors:** Altayeb Abdalla Ahmed, Rawia Mohamed Ahmed, Ahmed Jamleh, Gianrico Spagnuolo

**Affiliations:** 1 Department of Basic Medical Sciences, College of Medicine, King Saud Bin Abdulaziz University for Health Sciences, Riyadh 11481, Saudi Arabia; 2 King Abdullah International Medical Research Center, Riyadh 11426, Saudi Arabia; aojamleh@gmail.com; 3 Anatomy Department, Faculty of Medicine, University of Khartoum, Khartoum 11111, Sudan; rawia.omer8@gmail.com; 4 Restorative and Prosthetic Dental Sciences, College of Dentistry, King Saud Bin Abdulaziz University for Health Sciences, Riyadh 11481, Saudi Arabia; 5 Department of Neurosciences, Reproductive and Odontostomatological Sciences, University of Naples Federico II, 80131 Naples, Italy; gspagnuo@unina.it

**Keywords:** anterior loop, accessory mental foramina, cone-beam computed tomography, mandibular canal, mental canal, mental foramen

## Abstract

This study investigated the cone-beam computed tomography (CBCT)-based features of the mandibular canal, mental foramen, anterior loop, and accessory mental foramina with respect to age and sex. A total of 306 CBCT mandibular images were included in this retrospective study to measure the mandibular canal location and extension, the mental foramen position, the presence of the anterior loop, and the accessory mental foramina. The measurements were obtained in sagittal, coronal, and axial views. Descriptive statistics are presented. Sex-related differences, correlations, and comparisons were calculated using SPSS at 5% significance level. The mandibular canal was located more coronal and medial in male patients. The majority of cases had the mental foramen located just apical to the mandibular second premolar with a mean height of 2.94 mm and a mean length of 3.28 mm. Age affected the size of the mental foramen. The mental canal in all cases tended to show a coronal direction. Mesial extension of the anterior loop was found in 66.01% of the images while accessory mental foramina were detected in 2.6%. The complexity of the mandibular canal, mental foramen, anterior loop, and accessory mental foramina among Sudanese patients with respect to age and sex was confirmed.

## 1. Introduction

A thorough understanding of the complexity of mandibular canal (MC) anatomy, the inferior alveolar nerve (IAN) course, and the mental region is paramount for the safe conduct of root canal treatment and oral and maxillofacial surgeries in the mandible [1,2]. The IAN runs as a part of the inferior alveolar neurovascular bundle within the osseous MC. The MC is usually located close to the lingual surface of the bone till it reaches the mesial surface of the mandibular first molar, from where it becomes more buccally located until its termination in the mental foramen (MF) [3]. The mental nerve most commonly emerges from the MC in the anteroposterior direction, where it may form the anterior loop (AL) [2,4,5,6,7]. The prevalence and extension of the AL are variable among populations [5,8,9,10].

Although the MF is usually singular, accessory buccal openings may be present, known as accessory mental foramina (AMF) which show continuity with the MC [11]. These AMF contain accessory mental arteries and/or accessory mental nerves [12]. The existence of AMF affects the dimensions of the MF [13]. The prevalence of AMF varies between populations, with a range between 2% and 14.3% [2,11,14,15,16,17,18,19,20].

Various radiological methods have been used to properly assess the IAN course, MC, AL, MF, and AMF. Panoramic radiography used to be the standard method for preoperative diagnosis or implant planning. Nevertheless, previous reports indicated that this 2D image may result in errors in the estimation of actual anatomical sizes due to the magnification effect, which can cause over- or under-estimation. Furthermore, this radiograph provides limited information about the buccolingual position of the canal. Recently, cone-beam computed tomography (CBCT) has gained popularity as a reliable dental tool due to its accuracy in distance measurement and the potential for its use in three-dimensional analyses which make it more suitable for assessing fine mandibular anatomical details [6,21].

Since population-specific information is important for dental practitioners, this study was performed to assess the CBCT-based features of MC, MF, AL, and AMF among Sudanese patients with respect to age and sex. In addition, bilateral cases were compared.

## 2. Materials and Methods

### 2.1. Subjects

This retrospective study received approval from the Ethics Committee. Mandibular CBCT scans for 391 adult patients (older than 17 years old) who attended one dental radiographic center from June 2016 to February 2018 were retrieved. Any patient with evidence of bone disease, history of drug consumption that could affect the area, trauma or surgical history to the region, congenital disorders, a partially erupted mental region or unerupted teeth, pathologies at the mandibular premolars or molar areas, low image scan quality or artifacts, or images that did not show the mandible inferior border was excluded. Based on these exclusion criteria, 306 CBCT scans from 221 patients (85 of them had bilateral scans) were included.

### 2.2. Data Collection, Image Reconstruction, and Assessments

The CBCT data were collected using Planmeca ProMax3D (PLANMECA OY, Helsinki, Finland). The field of view was 15 × 12 cm and the voxel size was 0.200 mm. Scans were performed at 84 kV and 12–16 mA. All images were analyzed using Planmeca-Romexis (version 3.8.2.R) on a computer screen. The constructions and measurements were conducted on a 22-inch flat-panel color-active matrix thin-film transistor (TFT) medical display (S22F350FHM, Samsung, Shenzhen, China) with a resolution of 1920*1080 at 60 Hz under dim lighting conditions. All mandibles were assessed using images in three planes (axial, sagittal, and coronal) in addition to panoramic images. When necessary, 3D images were reconstructed.

**Figure 1:**
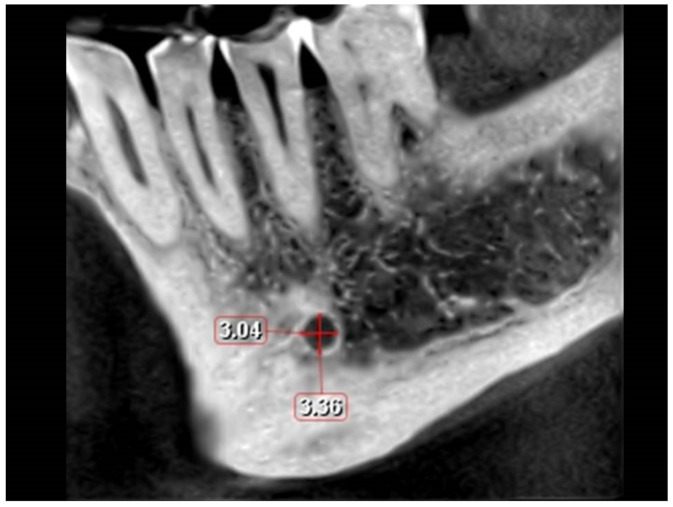
Height and length of the mental foramen (sagittal view).

**Figure 2:**
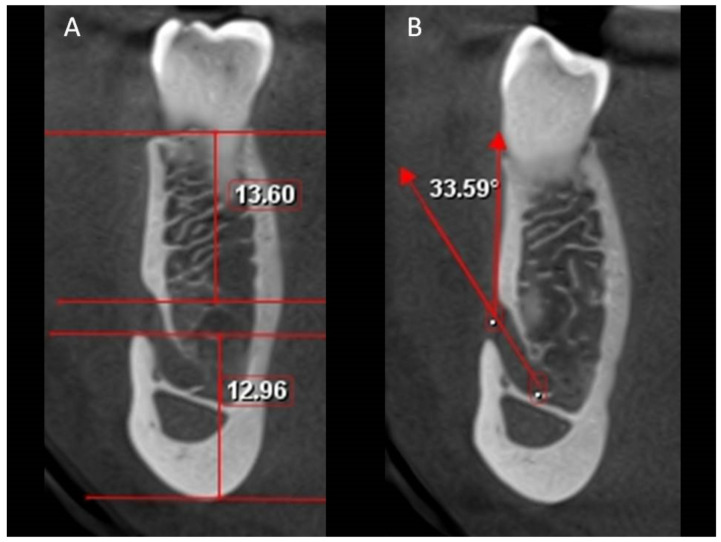
Coronal view showing the distance from the mental foramen to the upper and lower border of the mandible (**A**) and angulation between the mental canal and the buccal cortical bone superior to the mental foramen (**B**).

**Figure 3:**
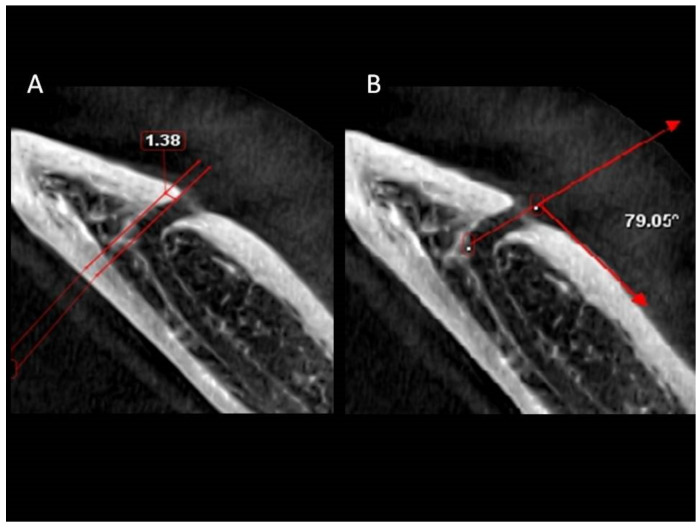
Axial view showing the mesial extension (**A**) and angulation between the mental canal and the buccal cortical bone posterior to the mental foramen (**B**).

Based on previous studies [2,6], the buccolingual location of the MC in the coronal plane, the position of the MF, the MF dimensions in the sagittal view (Figure 1) and the angle between the long axis of the mental canal (MeC) and the superior cortical buccal bone (Figure 2A,B), the presence of AL (Figure 3A,B), and the number of mental foramina in each scan were analyzed.

All measurements were repeated three times at an interval of at least one week to minimize measurement bias. Initially, 15 CBCT measurements were carried out by two observers. The intra- and inter-observer errors for measurements were not significant (*p* > 0.05).

### 2.3. Statistical Analysis

The data were analyzed using SPSS 21.0 (IBM Corp., Armonk, NY, USA). Descriptive statistics were presented. Independent t-test, Spearman rank correlation, one-way ANOVA, and Wilcoxon signed-rank test were used at a 5% significance level.

## 3. Results

Male patients constituted 43.4%. The patients’ mean age was 36.9 years (Range: 18–74 years). The numbers of patients included were 57, 88, 50, and 26 in the age groups of ≤25 years, 26–40 years, 41–55 years, and ≥56 years, respectively.

This study showed significant sex-related differences in the location of the MC in the regions of the mandibular second premolar and mandibular first and second molars (*p* < 0.001), with males tending to show a more coronal course than females in relation to the three teeth (Table 1). The MC tends to have a more lingual course in males than females in the mandibular first, second molar regions and mandibular second premolar region; however, these trends were not statistically significant.

A significant correlation exists in the mandibular second premolar-lingual (SPM-L) with age in male patients (r = 0.291, *p* = 0.025), whilst in female patients, the inferior distances in the mandibular molar regions were significantly correlated with age (mandibular first molar-inferior (FM-I): r = 0.165, *p* = 0.029 and mandibular second molar-inferior (SM-I): r = 0.181, *p* = 0.016). The dimension and location of the MF showed no significant correlation in males, whilst in females, a significant correlation was observed between MF location and mandibular first molar-lingual (FM-L) (r = 0.217, *p* = 0.004), FM-I (r = 0.170, *p* = 0.024), and SM-I (r = 0.156, *p* = 0.039).

Table 2 shows the position of the MF. The most common position was apical to the mandibular second premolar. No MF was observed anterior to the mandibular first premolar or between the mandibular first and second molars. There was no difference in the position of the MF according to sex or age (*p* > 0.05).

The size of the MF was assessed in the sagittal view (Table 3 and Table 4). The mean height was 3.14 mm among males and 1.64 among females (Range: 1.06–5.44 mm) and the mean length was 3.57 mm and 1.19 mm in males and females respectively (Range: 1.12–6.40 mm), with males showing significantly greater values than their female counterparts (*p* < 0.001). Also, age had a significant influence on length (*p* = 0.001). However, the MF position does not influence the MF dimensions (*p* > 0.05).

In the coronal view (Table 3 and Table 4), the mean distance between the superior margin of the MF and the mandible alveolar crest was 14.21 mm in males and 13.48 in females (Range: 8.96–19.36 mm), whereas the mean distance between the inferior margin and the lower border was 13.58 mm and 12.06 mm in males and females respectively mm (Range: 8.96–19.36 mm). Males showed significantly larger values than females for both distances (*p* < 0.01). Age shows a statistically significant influence only on the distance between the inferior margin and the lower border of the mandible (*p* < 0.01). The MF position affects only the distance between the superior margin of the foramen and the alveolar crest (*p* = 0.013).

In the coronal view, the angle between the MeC and the cortical bone surface was found to be coronal to the MC, with a mean angle of 46.67° in males and 13.48 mm in females (Range: 10.46–88.48°). This angulation is affected by sex (*p* = 0.009) and age (*p* = 0.008) but not location (*p* = 0.164) (Table 3 and Table 4). In the axial view, when assessing the angle between the MeC and the cortical bone surface distal to the MF, the mean angle was 76.40° in males and 77.65 mm in females (range, 30.75–128.18°) with no influence by sex, age, or location (*p* > 0.05).

The mesial extension of the AL was identified in 202 images (66.01%). 44.45% of the cases had an AL extension less than 2 mm and only 1.63% showed extensions exceeding 4 mm. The mesial extension was not influenced by location, sex, or age (*p* > 0.05).

Table 5 illustrates that the bilateral cases showed no statistically significant differences in terms of the MF measurements in the sagittal view, the distance between the superior margin of the MF and the crest of the alveolar bone in the coronal view, the distance between the inferior margin of the MF and the mandibular inferior border in the coronal view, the angle between the MeC and the buccal surface of the mandible in the coronal and axial views, and the AL extension (*p* > 0.05). Forty percent of the patients showed side asymmetry in the position of MF.

The most common MF pattern was a single MF (96.7%). AMF was found in nine cases with double foramina, and one case with triple foramina.

## 4. Discussion

This study assessed the IAN course within the MC and the associated anatomical variations. Among Sudanese, the largest distances between the MC and the inferior border of the mandible were observed at the mandibular premolar region followed by the regions of the mandibular first and second molars which match the findings in Asian populations [1,22,23]. Moreover, the canal position was located more coronal in males than in females similar to Malaysians and Spaniards [1,2]. The buccolingual relationship of the MC showed the greatest distance from the buccal bone at the mandibular second molar, followed by the mandibular first molar and mandibular second premolar. These findings agreed with previous reports in which the MC course showed a gradual lingo-buccal deviation in the posterior-anterior direction in Chinese, Iranians, and Malaysians [1,22,23]. Sudanese females tended to have a more laterally located MC, especially at the mandibular first and second molars, as in Spaniards [2]. In males, the only dimension that was correlated with age was the SPM-L (r = 0.291, *p* = 0.025). In females, the inferior dimensions in both molar regions were significantly correlated with age (FM-I: r = 0.0165, *p* = 0.029; SM-I: r = 0.181, *p* = 0.016). This finding is similar to the findings in Malaysians [1]. Clinically, these findings are important to avoid neurosensory sequelae in mandibular surgeries and to prove that although there is a general pattern in the MC spatial relationships, the safety zones do not show a universal pattern. Moreover, the findings also suggest that age and sex should be considered while planning surgeries in the mandibular region.

The current findings showed that the MF was most commonly located apical to the mandibular second premolar, followed by location between the two mandibular premolars. This is in agreement with populations having mixed ethnicities including Saudi, Jordanian, Egyptian, Moroccan, Indian, Malay and Chinese populations [7,9,18,24,25]. However, in other studies, the MF was most commonly located between the mandibular premolars, followed by area apical to the mandibular second premolar tooth, as in Northern Jordanian, Caucasians, Spaniards, and Swiss populations [2,5,6,15]. In 3.6% of cases, the foramen was apical to the mesial root of the mandibular first molar, contradicting the finding in a Swiss population, wherein no MF could be detected below the first mandibular molar [6]. Previous studies among sub-Saharan Africans showed that the MF was most commonly located between the second premolar and first molar, followed by a location in line with the second premolar in Zambians and Nigerians [26], while the most common position in Tanzanians, Malawians, and South-East Nigerians [27,28,29] was apical to the second premolar followed by location between the second premolar and the first molar. The variability in the MF position can be attributed to genetic factors, which is indicated by the general pattern of Africans showing mental foramina positioned more anteriorly than Europeans [30]. In this study, 40% of the bilateral images show side asymmetry in the position of MF, which is consistent with previous reports showing craniofacial asymmetry among Sudanese [31] and needs to be investigated further. Therefore, observance of these variations is extremely important to avoid patient injuries.

When considering MF dimensions, males showed greater values than their female counterparts. Significant sex-related differences were found in both height and length of MF. In contrast, while the length shows significant age-related differences, the height of MF lacks age-related differences. These differences are consistent with the findings among Turks [16]. In contrast, other studies reported no significant differences in the MF length in terms of age and sex [6,32]. Consistent with other studies, this study showed no bilateral asymmetry in MF dimensions [6,16].

The distances between the MF and the borders of the mandible were found to be affected by age and sex. The MF in Sudanese was found to be located more inferiorly than that in the Swiss population [6]. However, in both studies, males showed greater values than females. Among Sudanese, age shows a significant influence solely on the distances between the lower margin and the mandibular inferior border (*p* = 0.005). The effects of age on dimensions vary across populations, with a Swiss population showing no significant effect (*p* = 0.170) while a Spanish population showed a higher distance between the canal and the superior mandibular border in the younger patients [6,33]. These variabilities between populations can be attributed to craniofacial directional growth differences governed by racial and ethnic backgrounds and, to a lesser extent, by environmental and dietary habits.

The emergence of the MeC in the coronal view was found to show a coronal direction from the MC in all cases which is significantly affected by age and sex but not the location of the MF. Previous reports revealed the same direction with some cases showing a straight course in elderly patients [6,33]. In the axial view, age and sex had no effect on the MeC angulations which is consistent with the Swiss population [6]. In both studies, the age, sex, and location of the MF did not affect the angulations.

The AL length and extension have clinical importance in determining the safety zone for dental implantation in the inter-foraminal region. The AL was identified in about two-thirds of the examined Sudanese patients. Its presence varies among populations with a range of 15.2–96% [2,4,5,6,7,8,34,35]. These variabilities in prevalence may be due to differences in methodologies, ethnic backgrounds, or sample sizes, and age biases. In addition, the AL length was found to be within the range of previously published studies [4,5,9,34,35]. The AL mesial extension was not statistically influenced by location, sex, or age. Almost half of the cases showed less than 2 mm of mesial extension. Previous studies suggested that the safety zone anterior to the MF is 2, 3, or 5 mm during implant placement [4,21]. However, our study showed that in some cases, this may result in AL injury. Therefore, CBCT monitoring before these procedures is recommended to avoid iatrogenic injuries, and in the absence of this modality, a safety zone of 8 mm should be considered.

Identification of the AMF is important for adequate local anesthesia and to avoid nerve injuries and hemorrhages during surgical procedures [19]. In this study, the AMF was evident in only 2.6%, which is consistent with Asian Indians, White Americans, Saudis, and Spaniards, and African Americans [2,14,18]. In contrast, the presence of AMF was higher than 7% but not exceeding 12% in Northern Jordanians, Japanese, Turks, and Southern Chinese populations [11,15,16,17,19,20]. Furthermore, our finding shows that the AMF was slightly more common in females, but the difference was not statistically significant. The relationships between sex and the prevalence of AMF showed contradicting results based on the population and the technique adopted. For example, one study conducted among Turks indicated that AMF was not affected by sex, while another study showed it was more common in females [16,19]. Another report indicated that among Africans and Americans, it was more common in males [14]. The only case of multiple mental foramina in our study was observed in a male participant, in contrast to the findings in Turks, among whom all similar cases involved female participants [19].

The MC and MF are clinically important for their neurovascular content and their use as landmarks for anesthesia. MF injuries are associated with neurosensory disturbances in 8.5–24% of the cases for 6 to 16 months following surgery in the region [21,33]. The prevention of such disturbances should not depend on probabilities but should rather be based on prior knowledge and proper investigations.

In recent years in the field of oral and maxillofacial radiology, CBCT imaging discovery formed a significant technical advancement that revolutionized pertinent clinical practice. Conventional CT scans use a flat beam from a high-output X-ray source while CBCT scans utilize a cone radiation beam from an X-ray source. CBCT proved to be better than conventional CT due to its relatively lower cost, fewer space requirements, faster image acquisition with a larger volume, superior spatial resolution, and lower radiation exposure [2,36]. Therefore, the CBCT 3D-gathered information becomes complementary to other radiological modalities. However, CBCT imaging has its shortcomings and disadvantages which can be attributed to the selection of acquisition modes aiming for patient safety through a reduction in radiation dosages and/or the structural large cone geometry of the CBCT system. Among these is the possibility of the existence of image artifacts especially following metal restoration, the lack of ability to precisely depict the soft tissue’s internal structure, and reduced accuracy of the assigned Hounsfield unit (HU) used for standardized quantification of bone density [37]. It is worth mentioning that nano-structured radiation shielding materials are effective in the attenuation of X-ray energy [38].

Orthopantomogram was recommended as an initial investigation to predict IAN injuries following extraction surgery for lower impacted wisdom teeth while CBCT scans were considered by many practitioners as a keystone in pre-implantation assessment to plan minimally invasive surgery, conduct mocks for planned surgeries, and avoid iatrogenic injuries [39,40]. Also, CBCT can be used to assess the skeletal deformation in congenital diseases [41] This study emphasizes the importance of using the appropriate imaging modality on an individual basis to locate these structures before performing implantation or other surgical procedures related to the mandible. Furthermore, population-specific data are important, since the use of standards proposed for other populations reported in textbooks or journals can result in serious injuries and/or reduce the management efficacy.

The CBCT imaging usage in research is governed by strict regulations from Institutional Ethics Committees which require anonymity of data to ensure confidentiality and privacy; moreover, it is unethical to request CBCT for prospective researches [42].

## 5. Conclusions

The complexity of MC, MF, AL, and AMF among Sudanese patients with respect to age and sex was confirmed. The bilateral comparison lacks significant differences in these parameters. The mental foramen location differs in 40% of bilateral cases. These should be considered when planning for endodontic and surgical interventions.

## Figures and Tables

**Table 1:** The distance (in mm) between the mandibular canal and cortical plates in three directions (Buccal, lingual, and inferior) measured at the mandibular second premolar and the mandibular first and second molar regions.

Variable	Male		Female		*p*-Value
	Mean	Range	Mean	Range	
SPM-B	3.57 ± 1.33	1.00–8.00	3.32 ± 0.96	1.00–6.00	0.222
SPM-L	3.47 ± 1.31	1.28–6.56	3.80 ± 1.40	1.28–7.36	0.149
SPM-I	8.06 ± 1.55	4.48–11.52	6.92 ± 1.68	3.52–10.72	0.000 *
FM-B	4.51 ± 1.51	1.60–14.16	4.26 ± 1.13	2.24–6.88	0.105
FM-L	2.41 ± 1.13	0.64–6.09	2.49 ± 0.94	0.91–5.77	0.529
FM-I	7.51 ± 1.69	3.52–12.80	6.09 ± 1.61	2.72–10.88	0.000 *
SM-B	5.48 ± 1.56	1.76–10.56	5.26 ± 1.33	2.25–9.13	0.170
SM-L	2.04 ± 0.97	0.32–7.06	2.21 ± 0.92	0.64–5.77	0.111
SM-I	6.71 ± 1.66	3.20–11.36	5.57 ± 1.56	2.12–10.24	0.000 *

SD: Standard Deviation. SPM: Second premolar, FM: First molar, SM: Second molar. B: Buccal distance, L: Lingual distance, I: Inferior distance. The asterisk indicates statistical significance.

**Table 2:** Frequency analysis of the location of mental foramen.

Location	Number	Percentage (%)
Apical to the first premolar	6	2
Between the premolars	60	19.6
Just mesial to the second premolar	49	16
Apical to the second premolar	124	40.5
Between second premolar and first molar	56	18.3
Apical to the mesial root of the first molar	11	3.6
Total	306	100

**Table 3:** Analysis of the dimensions (in mm) and distance (in mm) of the mental foramen, angulation of the mental canal, and extension of the anterior loop on both sexes (*n* = 306 images).

Variable	Male (*n* = 136)				Female (*n* = 170)				Independent t-Test	
	Min	Max	Mean	SD	Min	Max	Mean	SD	t-Test	*p*-Value
Sagittal view										
Height of MF	1.66	5.44	3.14	0.72	1.06	4.48	1.64	0.70	4.463	0.000 *
Length of MF	1.60	6.40	3.57	0.96	1.12	5.65	1.19	0.78	5.358	0.000 *
Coronal view										
Angulations of MeC	17.51	87.39	46.67	14.82	1.06	4.48	42.38	13.82	2.616	0.009 *
Distance from MF to crestal bone	8.96	20.81	14.21	2.46	1.12	5.65	13.48	2.20	2.759	0.006 *
Distance from MF to lower border of mandible	9.28	19.36	13.58	1.78	1.06	4.48	12.06	1.35	8.231	0.000 *
Axial view										
Angulations of MeC in axial view	39.63	127.76	76.40	16.64	30.79	128.18	77.65	17.67	0.631	0.528
Mesial extension of AL	0.00	7.03	1.28	1.19	0.00	4.98	1.09	1.08	1.462	0.145

MF: Mental foramen. MeC: Mental canal. AL: Anterior loop. The asterisk indicates statistical significance.

**Table 4:** Analysis of the dimensions (in mm) and distance (in mm) of the mental foramen, angulation of the mental canal, and extension of the anterior loop on different age groups (*n* = 306 images).

Variable	Age (Years)	≤25	26–40	41–55	≥56	F Statistics	*p*-Value
		(*n* = 86)	(*n* = 123)	(*n* = 65)	(*n* = 32)		
Sagittal view							
Height of MF	Mean (SD)	3.02 (0.69)	2.81 (0.78)	3.05 (0.70)	3.03 (0.76)	2.351	0.072
	Range	1.68–4.80	1.06–5.44	1.66–4.96	1.92–4.80		
Length of MF	Mean (SD)	3.36 (0.78)	3.09 (0.89)	3.62 (0.98)	3.10 (0.90)	5.798	0.001 *
	Range	1.92–5.03	1.12–6.25	1.67–6.40	1.95–5.44		
Coronal view							
Angulations of MeC	Mean (SD)	42.28 (13.53)	42.65 (15.10)	49.44 (13.76)	45.49 (13.20)	4.038	0.008 *
	Range	10.46–87.39	16.08–88.48	21.22–84.93	21.64–78.54		
Distance from MF to crestal bone	Mean (SD)	13.70 (2.21)	14.04 (2.34)	13.51 (2.59)	13.77 (2.19)	0.825	0.481
	Range	9.61–21.61	8.80–20.81	8.96–20.55	9.61–17.77		
Distance from MF to lower border of mandible	Mean (SD)	12.19 (1.62)	12.86 (1.63)	12.99 (1.83)	13.18 (1.73)	4.414	0.005 *
	Range	9.28–17.31	8.96–18.56	9.28–19.36	9.44–17.12		
Axial view							
Angulations of MeC in axial view	Mean (SD)	74.59 (17.77)	78.32 (19.02)	77.10 (14.72)	79.13 (12.16)	0.965	0.410
	Range	35.69–127.76	30.79–128.18	45.67–116.85	58.74–107.87		
Mesial extension of AL	Mean (SD)	1.28 (1.28)	1.16 (1.17)	1.16 (0.98)	0.99 (0.83)	0.555	0.645
	Range	0.00–7.03	0.00–5.60	0.00–3.85	0.00–2.36		

MF: Mental foramen. MeC: Mental canal. AL: Anterior loop. SD: Standard deviation. The asterisk indicates statistical significance.

**Table 5:** Analysis of the dimension (in mm) and distance (in mm) of the mental foramen, angulations of the mental canal, and extension of the anterior loop in bilateral cases (*n* = 85).

Variables	Right Side			Left Side			*p*-Value
	Mean	Median	Range	Mean	Median	Range	
Sagittal view							
Height of MF	2.93	2.88	1.66–4.96	2.89	2.99	1.44–5.44	0.353
Length of MF	3.23	3.2	1.60–5.95	3.22	3.39	1.76–6.25	0.061
Coronal view							
Angulations of MeC	44.84	45.15	14.6–87.39	41.26	42.62	10.46–73.01	0.065
Distance from MF to the crestal bone	13.73	13.44	9.44–20.55	13.35	13.78	8.80–21.61	0.958
Distance from MF to lower border of the mandible	12.59	12.48	9.28–18.4	12.48	12.69	8.96–19.36	0.462
Axial view							
Angulations of MeC	78.43	76.26	39.63–128.18	76.48	76.68	36.94–127.76	0.766
Mesial extension of AL	1.19	1.13	0.00–7.03	1.15	1.09	0.00–5.60	0.570

MF: Mental foramen. MeC: Mental canal. AL: Anterior loop.

## Data Availability

The data presented in this study are available on request from the corresponding author.
